# Complete Genome Sequence of *Xanthomonas citri* pv. *anacardii* Strain IBSBF2579 from Brazil

**DOI:** 10.1128/genomeA.01574-17

**Published:** 2018-02-01

**Authors:** Wilson José Silva Junior, Antonio Roberto Gomes Farias, Nelson Bernardi Lima, Ana Maria Benko-Iseppon, Flávia Aburjaile, Valdir Queiroz Balbino, Raul Maia Falcão, Sérgio de Sa Leitão Paiva Júnior, Lucas Christian Sousa-Paula, Rosa Lima Ramos Mariano, Elineide Barbosa Souza, Marco Aurélio Siqueira Gama

**Affiliations:** a Department of Agronomy, Federal Rural University of Pernambuco, Recife, Brazil; b Department of Genetics, Federal University of Pernambuco, Recife, Brazil; c Department of Biology, Federal Rural University of Pernambuco, Recife, Brazil

## Abstract

The bacterium *Xanthomonas citri* pv. *anacardii* is the agent of angular leaf spot of the cashew tree (*Anacardium occidentale* L.). The complete genome sequencing of the strain IBSBF2579 was done on an Illumina HiSeq 2500 platform. The *de novo* assembly of the *X. citri* pv. *anacardii* strain IBSBF2579 genome yielded 133 contigs, with a size of 5,329,247 bp and a G+C content of 64.03%. The prediction was performed by GeneMarkS and the automatic annotation by Rapid Annotations using Subsystems Technology (RAST), with 4,406 identified genes.

## GENOME ANNOUNCEMENT

The cashew tree is native to Brazil (1) and grows in South America’s tropical regions, mainly in the northeast of the country (2). Cashew nut production may be impaired by the occurrence of diseases, such as standout angular leaf spot caused by *Xanthomonas citri* pv. *anacardii* and *X. citri* pv. *mangiferaeindicae* (3, 4). These bacteria show pathological differences in cashew and mango trees, being able to multiply and survive in different hosts in the Anacardiaceae family (5, 6). However, until now, only *X. citri* pv. *mangiferaeindicae* (LMG 941) has been sequenced (7). Here, we report the whole-genome sequence of the type strain of *X. citri* pv. *anacardii* (IBSBF2579 = ICMP4088), which is nonpigmented and was isolated from a cashew tree in Brazil. The genome sequencing was performed by using the Illumina HiSeq 2500 platform at the Functional Genomics Center of the University of São Paulo. The libraries were prepared with the Illumina Nextera XT DNA library prep kit v 4, and sequencing was performed on a HiSeq flow cell v4, with the HiSeq SBS kit v4 and paired reads of 100 bp (2×). Initially, the quality reads were analyzed by FastQC (8), and read trimming to remove the adapters was performed by using FASTX-Toolkit (v. 0.0.13) (9). The genome *de novo* assembly was carried out using SPAdes (v. 1.10) (10), yielding 133 contigs of >500 bp (*N*_50_, 150,594 bp), with the largest contig being 344,686 bp, for a total assembly size of 5,329,247 bp with a G+C content of 64.03%. The annotation using GeneMarkS (11) predicted 4,406 genes by Rapid Annotations using Subsystems Technology (RAST) (12). All assembly statistics were generated with QUAST (v.4.6) (13).

### Accession number(s).

This whole-genome shotgun project has been deposited at DDBJ/ENA/GenBank under the accession number PESI00000000. The version described in this paper is version PESI01000000.
